# Covalent Protein Modification with ISG15 via a Conserved Cysteine in the Hinge Region

**DOI:** 10.1371/journal.pone.0038294

**Published:** 2012-06-05

**Authors:** Veronika N. Bade, Jochen Nickels, Kirstin Keusekotten, Gerrit J. K. Praefcke

**Affiliations:** Center for Molecular Medicine Cologne (CMMC), Institute for Genetics, University of Cologne, Cologne, Germany; University of Hong Kong, Hong Kong

## Abstract

The ubiquitin-like protein ISG15 (interferon-stimulated gene of 15 kDa) is strongly induced by type I interferons and displays antiviral activity. As other ubiquitin-like proteins (Ubls), ISG15 is post-translationally conjugated to substrate proteins by an isopeptide bond between the C-terminal glycine of ISG15 and the side chains of lysine residues in the substrates (ISGylation). ISG15 consists of two ubiquitin-like domains that are separated by a hinge region. In many orthologs, this region contains a single highly reactive cysteine residue. Several hundred potential substrates for ISGylation have been identified but only a few of them have been rigorously verified. In order to investigate the modification of several ISG15 substrates, we have purified ISG15 conjugates from cell extracts by metal-chelate affinity purification and immunoprecipitations. We found that the levels of proteins modified by human ISG15 can be decreased by the addition of reducing agents. With the help of thiol blocking reagents, a mutational analysis and miRNA mediated knock-down of ISG15 expression, we revealed that this modification occurs in living cells via a disulphide bridge between the substrates and Cys78 in the hinge region of ISG15. While the ISG15 activating enzyme UBE1L is conjugated by ISG15 in the classical way, we show that the ubiquitin conjugating enzyme Ubc13 can either be classically conjugated by ISG15 or can form a disulphide bridge with ISG15 at the active site cysteine 87. The latter modification would interfere with its function as ubiquitin conjugating enzyme. However, we found no evidence for an ISG15 modification of the dynamin-like GTPases MxA and hGBP1. These findings indicate that the analysis of potential substrates for ISG15 conjugation must be performed with great care to distinguish between the two types of modification since many assays such as immunoprecipitation or metal-chelate affinity purification are performed with little or no reducing agent present.

## Introduction

Interferons (IFNs) play a key role in the defence against viral, bacterial and protozoan infections [1]. The biological response to IFNs requires binding of the IFN molecules to type-specific receptors, which results in the activation of signalling pathways and the transcriptional upregulation of hundreds of IFN-stimulated genes (ISGs). One of the most highly induced ISGs by type I IFN (e.g. α and β) is ISG15 [2]. ISG15 is a critical molecule for the response against several viral infections since ISG15−/− mice are more sensitive to influenza A and influenza B viral infections and also show an increased susceptibility to murine herpes virus and to Sindbis virus [3]. The increased sensitivity of ISG15 knockout mice to Sindbis virus infection could be rescued by expressing wild type ISG15 [3]. Moreover, it has been shown that both, conjugated ISG15 and the unconjugated form possess antiviral activity [4]–[6].

ISG15 can also be secreted from human monocytes and lymphocytes [7] to act as a cytokine by inducing the production of IFN-β, proliferation of natural killer cells, neutrophil chemotaxis, and dendritic cell maturation [2], [8]–[10].

Initially, ISG15 has been identified as ubiquitin cross-reactive protein (UCRP) in mouse tumor cells [11] and it shares an amino acid sequence identity of about 30% with ubiquitin [12]. The overall structure of ISG15 consists of two ubiquitin-like domains each adopting a β-grasp fold that is nearly identical to ubiquitin. The two domains are connected by a six residue extended linker, the hinge region, which comprises the amino acids Asp76–Pro81 in human ISG15 including the water accessible Cys78 [13]. As many other Ubls, human ISG15 is expressed in an inactive precursor form. The maturation process is a proteolytic cleavage of the C-terminus in order to expose a di-glycine motif which is necessary for conjugation [12]. This reaction can be catalysed by the ubiquitin-related protease (UBP43) [14] but *in vivo* UBP43 seems to have also functions which are unrelated to ISG15 [15].The classical pathway of ISG15 conjugation (ISGylation) is initiated by the generation of a thioester between the C-terminal glycine of ISG15 and a cysteine of the activating E1 enzyme UBE1L. The activation energy is supplied by ATP. ISG15 is then transferred to Cys85 in the active site of the conjugating E2 enzyme UbcH8 (Ube2L6) via a thioester bond. Together with the E2, an ISG15 E3 ligase (e.g. Herc5, EFP or Hhari) transfers ISG15 to the ε-amino group of a lysine side chain in the substrate protein [16]–[20]. Besides ISG15 itself, also other components of the ISGylation cascade (UBE1L, UbcH8, the E3s and UBP43) are inducible by IFNs [17], [21]–[23]. As a result, the levels of ISG15 conjugates are tightly controlled by IFN. In several studies, hundreds of target proteins for ISGylation have been identified which are involved in diverse cellular pathways [4], [18], [20], [24]–[27]. Only some substrates were found in all studies which may be attributed to the fact that different cell lines were used [18], [20], [24]–[26]. Furthermore, some studies used IFN-induced cells [20], [24] while others transfected the proteins of the ISG15 conjugation cascade [18] or both [25]. The function of ISG15 modification has only been assessed for very few ISG15 substrates. One of them is Ubc13, which forms a heterodimer with Mms2 [28]. This complex has ubiquitin-conjugating activity and mediates the formation of K63-linked polyubiquitin chains on proteins involved in DNA repair, NF-κB signalling and mitosis [29]–[31]. Zou *et al*. have shown that modification of Ubc13 by ISG15 at Lys92 suppresses its ability to build a thioester bond with ubiquitin and thus Ubc13-mediated ubiquitylation is impaired [32]. Consequently, modification of Ubc13 by ISG15 has an indirect effect on different cellular functions. A negative regulation by ISGylation has also been reported for the oncogenic fusion protein of the promyelocytic leukemia protein (PML) and the retinoic acid receptor α (RARα) [33]. The authors reported that UBE1L mediated ISGylation of the PML part in the PML/RARα protein is involved in its proteasomal degradation. Consistent with this, depletion of the ISG15 specific protease USP43 destabilized the PML/RARα protein [34].One example of how ISG15 interferes with viral replication is the modification of viral proteins such as NS1 (Non-structural protein 1) from influenza virus A, which is ISGylated on Lys 41 [35]. ISG15 also serves as a negative feedback regulator of interferon induction by modifying the IFN-inducing protein RIG-I (Retinoic acid-inducible gene-I) [36]. ISG15 conjugation to RIG-I increases in the presence of a proteasomal inhibitor, which may indicate a crosslink between proteasomal degradation and ISGylation [36]. Other potential ISG15 substrates with antiviral function include the dynamin-like GTPases MxA (myxoma resistance protein 1) and hGBP1 (human guanylate binding protein 1) [25]. MxA confers resistance to influenza A virus and vesicular stomatitis virus [37], [38]. Zhao *et al*. have shown that MxA can be multiply modified by ISG15, but the function of this modification remained unknown [25]. Recently, Durfee and colleagues have found that newly synthesized proteins are predominant targets for ISGylation and that the ISG15 ligase Herc5 is associated with polyribosomes [39]. They propose an alternative model for the antiviral function of ISGylation by blocking the regular assembly of viral capsids.

We were interested to analyse the modification of several ISG15 substrates in more detail by applying a similar transfection and purification strategy as the above mentioned studies. In the course of our experiments, however, we observed that the levels of ISG15 modified proteins were increased in the presence of low amounts of reducing agents which are required for metal-chelate affinity purifications and immunoprecipitations. We demonstrated that this ‘atypical’ reducing agent sensitive ISG15 modification exists also in living cells and may be functionally relevant. Further investigations of the mechanism of ISG15 modification by mutational analysis and miRNA-mediated knock-down revealed that Cys78 in the hinge region of ISG15 is responsible for the atypical ISG15 modification and that Ubc13 can be modified via this cysteine residue.

## Results

### ISG15 Modification can be Decreased by Reducing Agents

**Figure 1:**
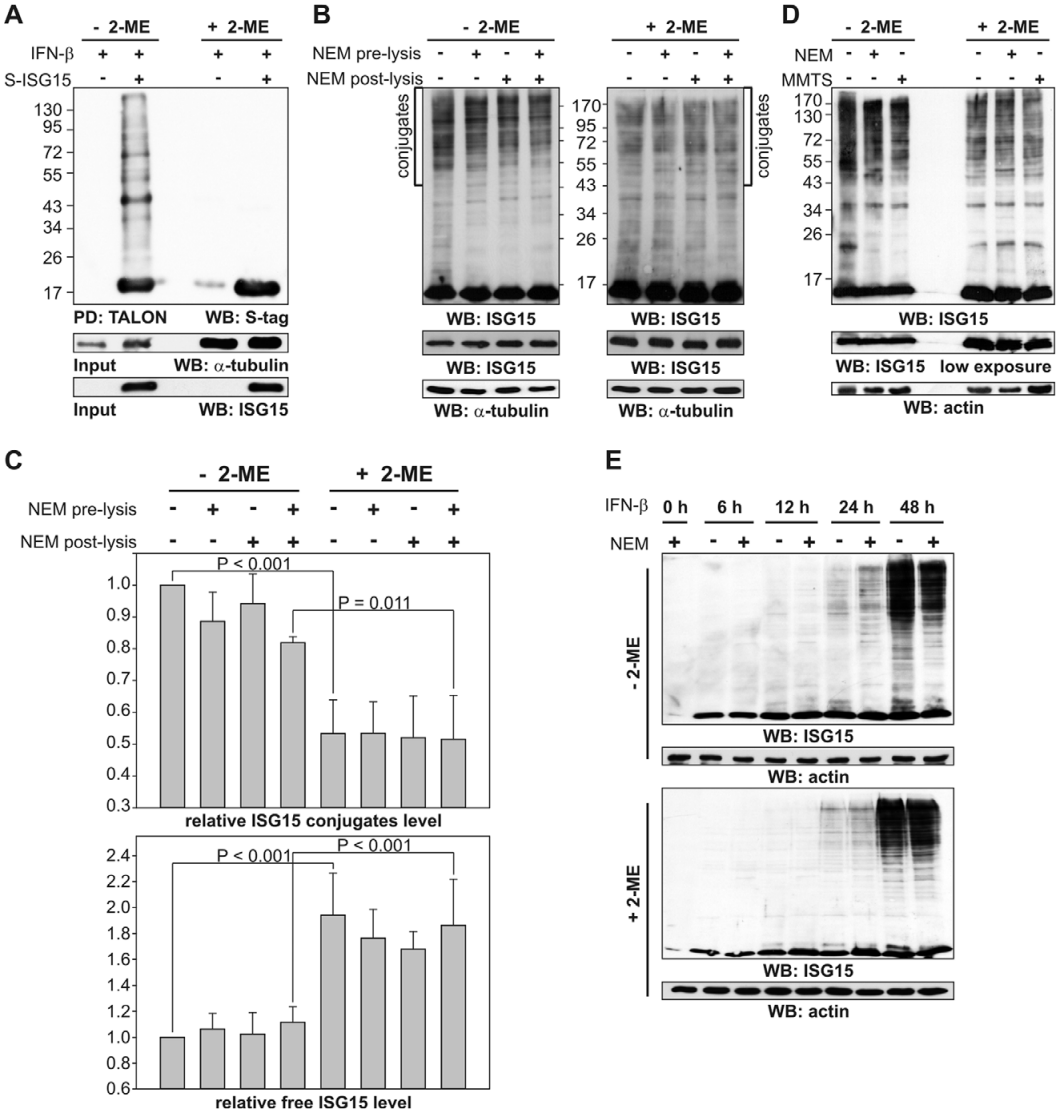
Decrease of ISG15 conjugates by reducing agents. (A) HeLa cells were transiently transfected with pTriEx2-His-S-ISG15. 24 h post-transfection, the cells were induced with IFN-β (1,000 units/ml). Purifications of ISG15 modified proteins were carried out under denaturating conditions without 2-ME. Eluates were equally split and treated with or without 2-ME before SDS-PAGE. (B) The IFN-β induced HeLa cells were treated with or without 40 mM NEM (NEM pre-lysis) for 30 min at 37°C. The cells were lysed with or without 20 mM NEM (NEM post-lysis). Equal loading of total protein is indicated by immunoblotting against alpha-tubulin. (C) The presence of reducing agent leads to a decrease of ISG15 modification and an increase of free ISG15. All data were derived from three independent experiments. Statistical analyses were conducted using SigmaPlot10 (Systat Software Inc) software and values were presented as mean ± SD. Significant differences between the groups were analysed by One Way ANOVA followed by Student-Newman-Keuls Method. A value of P<0.05 was accepted as an indication of statistical significance. (D) IFN-β induced HeLa cells were treated with or without NEM (pre- and post-lysis as in Figure 1B) and 1 mM MMTS (pre- and post-lysis). PVDF membrane was stripped and immunodecorated with anti-actin antibody (low panel). (E) The time course of ISG15 conjugation was studied after induction of HeLa cells with IFN-β for indicated times treated with or without NEM as in Figure 1D.

In order to analyse the modification of several IFN-induced ISG15 substrates, we established a protocol to isolate ISG15 modified proteins after IFN-β induction of HeLa cells transfected with RGS-His- or His-S-tagged maturated human ISG15 (residues 1–157) by metal-chelate affinity purification in analogy to the study by Zhao *et al.* [25]. Under these conditions, we expect that also considerable amounts of the presumed ISG15 processing and deconjugating enzyme UBP43, the activating and conjugating proteins UBE1L and UbcH8 and also several ligating enzymes such as Herc5, Hhari and EFP are expressed. In order to optimize the purification, we compared different metal-chelate matrices which work in the presence of different concentrations of reducing agents (DTT and 2-ME). When we analysed the amount of ISG15 modified proteins in the cell lysates and after metal-chelate purification by Western blotting, we observed that ISG15 modification was decreased with increasing amounts of reducing agent while free ISG15 became more abundant (data not shown). This effect became much more prominent, when the SDS-PAGE was performed with a loading buffer without reducing agent (Figure 1A). To avoid the formation of SDS resistant aggregates, it was important not boil the samples without reducing agent. A critical question arising from these results was if this type of ISG15 modification existed also in living cells or whether it was a mere artefact caused during cell lysis in buffers containing low amounts of reducing agents. Therefore IFN-induced HeLa cells were treated with N-ethylmaleimide (NEM) which is cell-permeable and irreversibly blocks free thiol groups [40]. NEM was added either 30 min before or during lysis or both and the levels of ISG15 modified proteins in the absence and presence of reducing agent were compared and analysed (Figure 1B). Addition of NEM led only to a slight reduction of ISG15 modification. In all cases the levels of ISG15 modification could be decreased by the addition of reducing agent while the levels of free ISG15 increased showing that the reduction sensitive modification had already existed in living cells. The reduction of ISG15 conjugates was readily detectable along the length of the SDS-PAGE from above 43 kDa indicating that many ISG15 substrates are affected. The quantification of the entire ISG15 signal in this region and of free ISG15 running approximately at 15 kDa indicated that about 40% of the total ISG15 modifications are sensitive to reducing agents (Figure 1C). Similar results were obtained with MMTS (Figure 1D) another thiol-blocking reagent [41]. Addition of hydroxylamine at neutral pH to cell lysates had no effect on ISG15 conjugates in the absence of 2-ME. This indicates that the reducing agent sensitive ISG15 conjugates are linked by disulphide bridges and not thioesters (Figure S1). A time resolved analysis revealed that ISG15 conjugates occur already 6–12 h after IFN induction but until 24 h post induction they are mainly composed of reduction sensitive conjugates (Figure 1E).

### Cys78 of ISG15 Plays a Role in Modification

**Figure 2:**
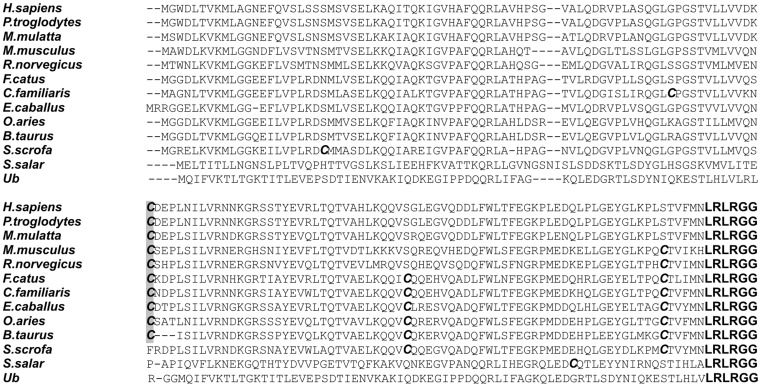
Amino acid alignment of ISG15 proteins from different vertebrate species with human di-ubiquitin. The potential functionally important sites are highlighted. The C-terminal site including the double glycine motif for the conjugation to substrates is marked in bold, cysteine residues are indicated in bold and italics and the conserved cysteine residues in hinge region are shaded in grey. Sequence analysis was performed using ClustalW.

Protein modifications which are sensitive to reducing agents include disulphide bridges and thioesters. While the amount of disulphide bridges within the cytosol is usually low due to the positive redox potential of the cytosol [42], thioesters occur as high energy compounds including the activated states of ubiquitin-like proteins with their activating E1 and conjugating E2 enzymes and with HECT-type E3 ligases. In these cases, specific cysteine residues in the active sites of the E1, E2 or E3 enzymes are linked to the carboxyl group of the C-terminal glycine residue in the Ubl. The only cysteine residue in human ISG15 (Cys78) is situated in the hinge region between the two ubiquitin-like domains. This cysteine is highly conserved in other mammalian ISG15 proteins with the exception of porcine ISG15 (Figure 2). Moreover, Cys78 in ISG15 has been found to be highly reactive leading to dimerization of the protein or to the modification by nitric oxide (NO) [43], [44].

**Figure 3:**
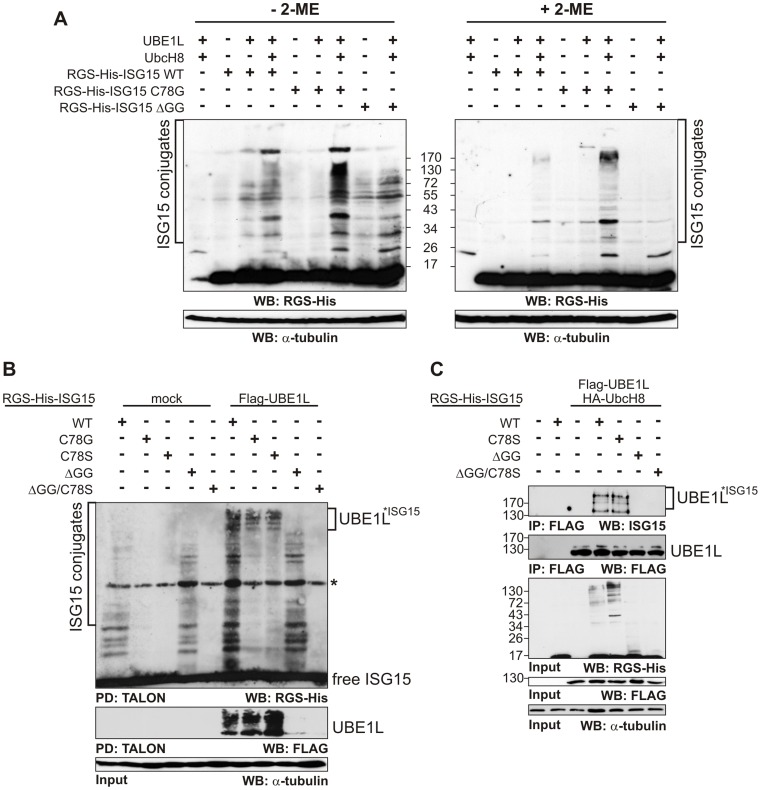
Mutational analysis of ISG15. (A) HeLa cells were transiently transfected with pCMV2a-Flag-UBE1L, pCMVb-HA-UbcH8 and pCMVb-MRGS-His-ISG15 WT or different mutants. 24 h post-transfection, the cells were collected and lysed with or without 2-ME. (B) Isolation of ISG15 conjugates by metal-chelate pull-down. HeLa cells were transiently transfected with pCMV2a-Flag-UBE1L and pCMVb-MRGS-His-ISG15 WT and different mutants. 24 h post-transfection the cells were collected and lysed without 2-ME. The metal-chelate pull-downs and the SDS-PAGE were carried out under denaturing conditions without 2-ME. The asterisk indicates an unspecific band. (C) Analysis of ISG15 modification of UBE1L by anti-FLAG pull-down. HeLa cells were transiently transfected with pCMV2a-Flag-UBE1L, pCMVb-HA-UbcH8 and pCMVb-MRGS-His-ISG15 WT and different mutants. 24 h post-transfection, the cells were collected and lysed. Flag-UBE1L was immunoprecipitated with anti-FLAG M2 Sepharose as described. SDS-PAGE was carried out in presence of 2-ME. Equal protein loading was verified by immunoblotting against alpha-tubulin.

In order to understand the nature of this atypical ISG15 modification, we generated mutants of maturated human ISG15 where Cys78 was either exchanged to glycine (ISG15-C78G), or to serine (ISG15-C78S) or where the C-terminal Gly-Gly sequence was deleted (ISG15-ΔGG) as well as a double mutant containing both mutations (ISG15-C78S/ΔGG). The levels of ISG15 modification of these mutants and ISG15-WT were analysed by Western blotting after co-transfection with Flag-tagged UBE1L and HA-tagged UbcH8 (Figure 3A) in non IFN-induced cells. ISG15-WT modification was increased by co-transfection with UBE1L and even further with UBE1L and UbcH8 but was sensitive to reducing agent. ISG15-C78G alone or with UBE1L displayed almost no ISG15 modification but high levels in the presence of both UBE1L and UbcH8. Under these conditions, ISG15 modifications in the low (26–43 kDa) and in the high (> 130 kDa) but not in the medium (43–130 kDa) molecular weight (MW) range were insensitive to reducing agents. ISG15-ΔGG mainly displayed modified proteins in the medium range (43–130 kDa) which were slightly increased by co-expression of UBE1L and UbcH8 but which were completely lost in the presence of reducing agents. Similar results were obtained by Co^2+^−chelate affinity purification of ISG15 modified proteins without 2-ME which also showed that no ISG15 modification occurred in the presence of the ISG15-C78S/ΔGG double mutant (Figure 3B). Anti-FLAG Western blotting revealed that the high MW bands of ISG15-WT, ISG15-C78S and ISG15-C78G are linked to UBE1L. Since the SDS-PAGE was performed without reducing agent, these high MW bands could represent ISG15 intermediates linked via thioester to UBE1L. We further investigated the role of Cys78 in the context of the modification of UBE1L. Immunoprecipitation of Flag-tagged UBE1L with subsequent SDS-PAGE under reducing conditions confirmed that UBE1L can be ISGylated by ISG15-WT and ISG15-C78S at multiple sites but none of the mutants lacking the double glycine were able to modify UBE1L, identifying UBE1L as a classically modified ISG15 substrate (Figure 3C).

### Ubc13 can be Modified by ISG15 via an Isopeptide Bond and a Disulphide Bond

**Figure 4:**
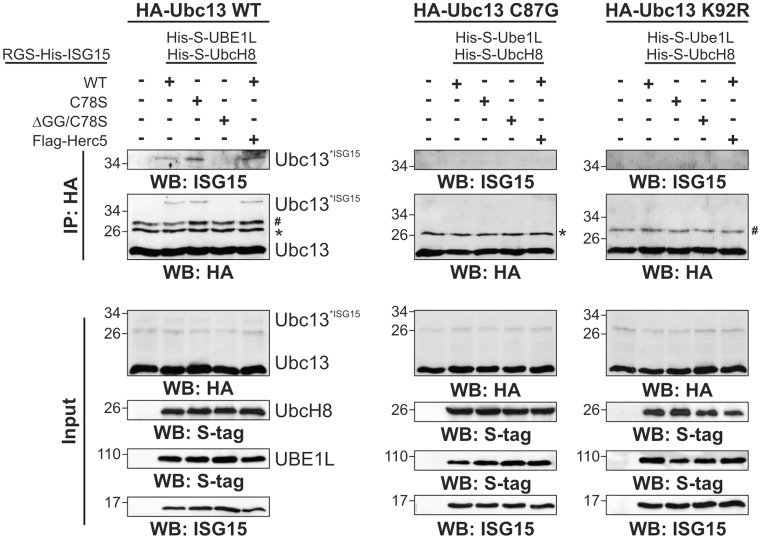
Different types of ISG15 modification of Ubc13. HeLa cells were transiently transfected with pCMVb-HA-Ubc13 WT, pCMVb-HA-Ubc13 C87G mutant or pCMVb-HA-Ubc13 K92R mutant and other vectors as shown in the figure. 24 h post-transfection the cells were collected and lysed. Anti-HA immunoprecipitations were performed as described. Immunoblotting against the S-tag shows the expression levels of both UBE1L and UbcH8. SDS-PAGE was carried out in presence of 2-ME. The asterisk indicates monoubiquitylated Ubc13 and the hash dimers of Ubc13.

Next we were interested to see whether other known ISG15 substrates are in fact not linked via an isopeptide bond but via the reducing agent sensitive linkage to Cys78. To this end, we transiently transfected Ubc13 which has been described as ISG15 substrate in the literature [24], [45] together with wild type or mutants of ISG15 and with UBE1L, UbcH8 and Herc5. As before for UBE1L we checked for ISG15 modification in the presence of reducing agent in total lysates and after immunoprecipitation of the substrate (Figure 4). After immunoprecipitations, Ubc13, which has a molecular weight of 17 kDa, ran at a major band at 17 kDa, two minor bands around 26 kDa and another weak band above 34 kDa. The bands at 26 kDa represent Cys-linked dimers of Ubc13 and monoubiquitylated Ubc13. Western blotting against ISG15 revealed that band above 34 kDa corresponds to ISG15 modification, since it was absent in the case of the ΔGG/C78S mutant and enhanced by transfection of the ISG15 ligase Herc5. The ISG15 conjugation to Ubc13 was highly reduced by the mutation of Cys87 in Ubc13 (see also further below) and completely abolished by mutation of the previously published ISGylation site K92.

**Figure 5:**
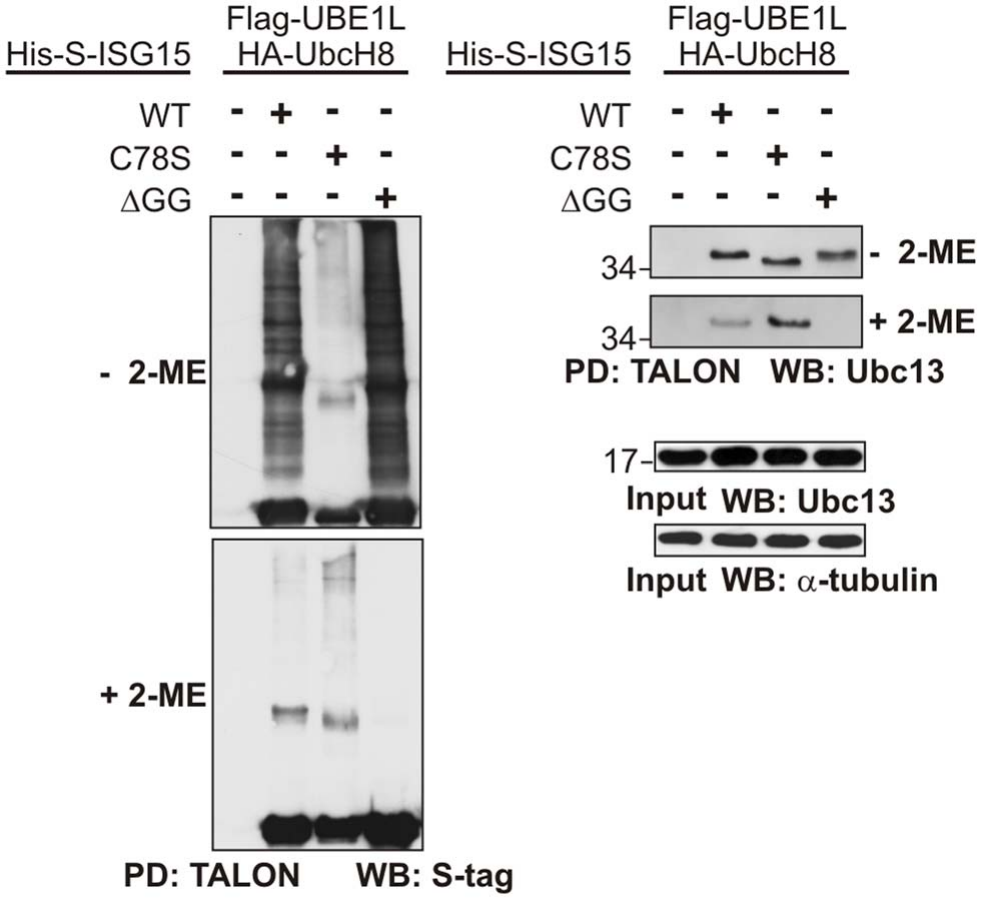
Different types of ISG15 modification of endogenous Ubc13. HeLa cells were transiently transfected with pCMVb-HA-Ubc13 WT (A) or pCMVb-HA-Ubc13 C87G (B) mutant and other vectors as shown in the figure. 24 h post-transfection the cells were collected and lysed in presence 2-ME. The metal-chelate pull-downs were carried out under denatured conditions with or without 2-ME. Immunoblotting against the S-tag show the levels of ISG15. Equal loading of total protein was verified by anti alpha-tubulin immunoblotting.

To examine whether ISG15 can be also linked via Cys78 to endogenous Ubc13, we performed a pull-down of ISG15 conjugates on Talon matrices with and without 2-ME (Figure 5). Therefore, HeLa cells were transiently transfected with His-S-ISG15 (wild type and mutants), Flag-UBE1L and HA-UbcH8. As before, we observed a strong effect of reducing agent on the total amount of ISG15 modifications, which was diminished in the case of the C78S mutant while the ΔGG mutant is able to take part in the formation of reduction sensitive ISG15 modifications. A stable isopeptide linkage to Ubc13 could be observed with the ISG15 Cys78 mutant and thus also confirmed previous results from others [32], [45]. Furthermore, endogenous Ubc13 is able to covalently interact with ISG15-WT as well as the C78S and ΔGG mutants without 2-ME (Figure 5). However, in the presence of 2-ME, the modification of Ubc13 with ISG15-WT is less pronounced and completely lost with the ΔGG mutant, indicating that at least parts of the interaction observed under non-reducing conditions is neither a thioester between the C87 in Ubc13 and the C-terminus of ISG15 nor a stable isopeptide linkage but instead occurs via Cys78 in ISG15.

### MxA and PML are not Modified by ISG15

**Figure 6:**
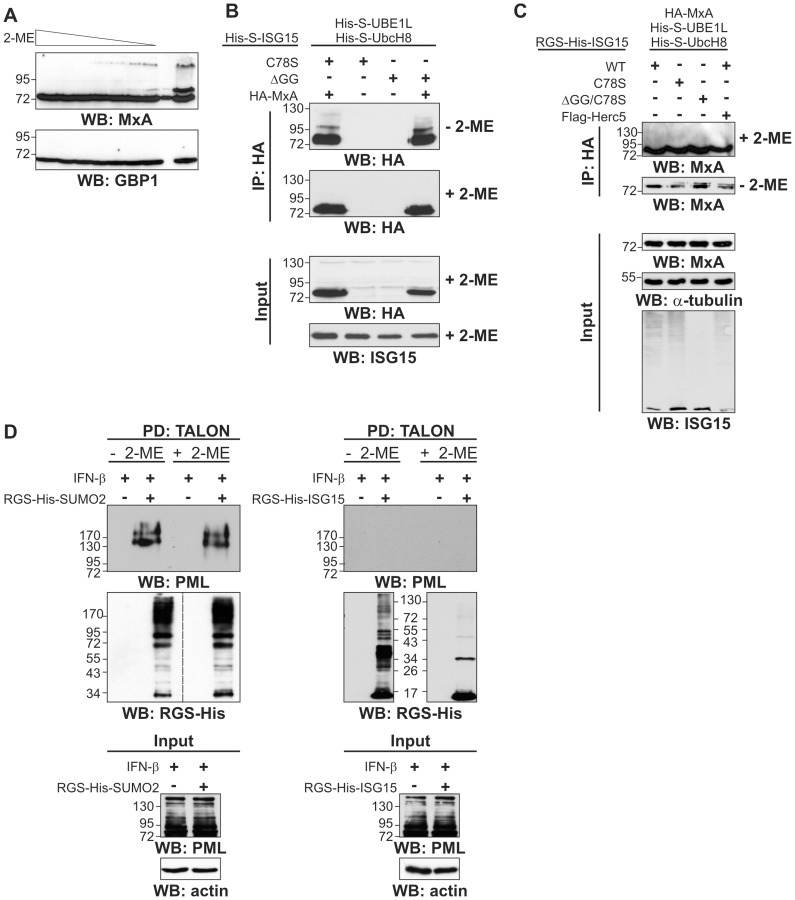
No evidence for ISG15 modification of MxA, hGBP1 and PML. (A) HeLa cells were induced with IFN-β for 24 h. The cells were lysed in urea buffer without reducing agent. Cellular lysates were equally aliquoted and 2-ME was added (loading to SDS-PAGE from left to right: 500 mM, 100 mM, 50 mM, 20 mM, 10 mM, 5 mM, 1 mM 2-ME, empty lane, 0 mM 2-ME) and blotted for MxA and hGBP1. (B) HeLa cells were transiently transfected with pCMV2b-Flag-MxA and the components of the ISG15 conjugation machinery as indicated in the figure. 24 h post-transfection, the cells were induced with IFN-β24 post-induction the cells were collected and lysed without 2-ME. Anti-FLAG immunoprecipitations were performed without 2-ME. Eluates were equally split and treated with or without 2-ME before SDS-PAGE (C) HeLa cells were transiently transfected with pCMVb-HA-MxA and components of the ISG15 conjugation machinery as indicated in the figure. 24 h post-transfection, the cells were collected and lysed without 2-ME. Anti-HA immunoprecipitations were performed. Eluates were equally split and treated with or without 2-ME before SDS-PAGE. (D) HeLa cells were transiently transfected with either pCMVb-MRGS-His-ISG15 or pCDNA4/TO/N-MRGS-His-SUMO2. 24 h post-transfection, the cells were induced with IFN-β (1,000 units/ml). Purifications of ISG15 or SUMO2 modified proteins were carried out under denaturating conditions without 2-ME. Eluates were equally split and treated with or without 2-ME before SDS-PAGE.

Similar to our previous experiments, we tried to investigate the ISG15 modification of MxA and also of the human guanylate-binding protein 1 (hGBP1) which have been described in the literature [25]–[26], [39] **.** In cell lysates of IFN-induced cells we couldn’t detect any slower migrating bands for hGBP1, while we observed several additional bands in Western blots against MxA in the range between 95–130 kDa. Those bands vanished with increasing concentrations of reducing agents in the SDS-loading buffer (Figure 6A). After immunoprecipitations of MxA these bands were not observed in the presence of 2-ME (Figure 6B, C), so that we assumed MxA is atypically modified by ISG15. The co-expression of WT or mutant ISG15, even with Herc5 had also no influence on the appearance of these bands (Figure 6B, C). In cases where these bands were detected, they displayed variable intensities between experiments and they were always sensitive to reducing agents. Together with the previous experiments we conclude that the higher MW bands of MxA are neither caused by classical nor atypical ISG15 modification, but probably appear due to running artefacts on SDS-PAGE with insufficient amounts of reducing agent and boiling. We also found no evidence for an ISG15 modification of the endogenous PML protein. In the same experiment we were able to specifically isolate PML, which was modified by the small ubiquitin-related modifier (SUMO) proteins (Figure 6D) [46], [47].

### Downregulation of ISG15 Modification by miRNA

**Figure 7:**
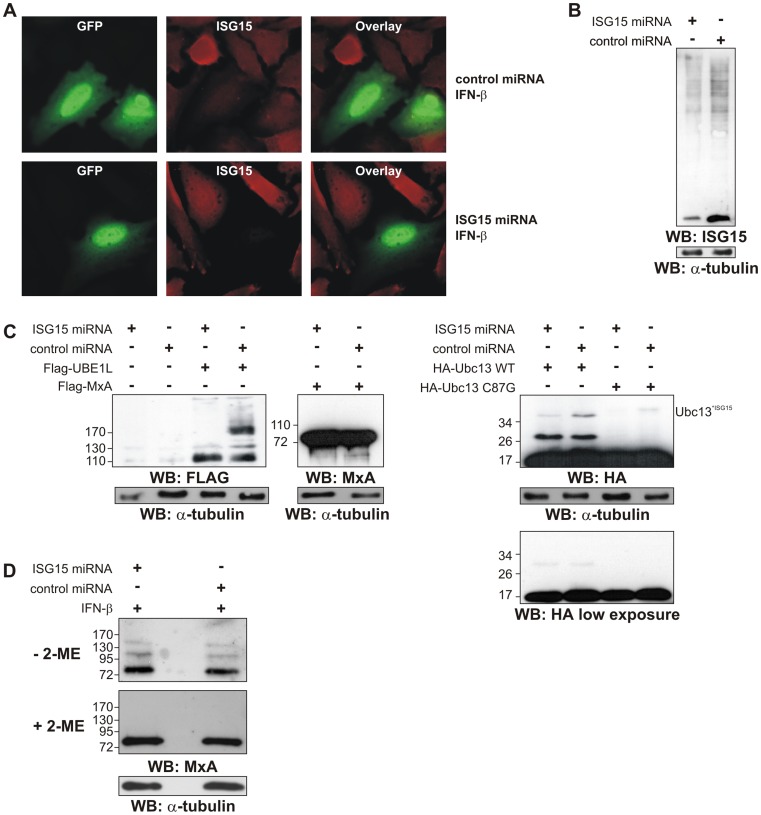
Substrate analysis after downregulation of ISG15. HeLa cells were transiently transfected with ISG15 or control miRNA vectors in which the miRNA is coded from the 3′-UTR of GFP. 12 h post-transfection the cells were induced with IFN-β for another 12 h. (A) After a total of 24 h post-transfection, cells were fixed and subjected to immunofluorescent staining with rabbit polyclonal ISG15 antibody and secondary Alexa Fluor 647 donkey-anti-rabbit antibody (red) and compared to the GFP signal (green). (B) GFP-positive cells were FACS sorted, lysed in urea buffer and analysed for ISG15 downregulation by Western blotting with reducing agent. (C) HeLa cells from the experiment in (B) were additionally co-transfected with pCMV2a-Flag-UBE1L, pCMV2b-Flag-MxA, pCMVb-HA-Ubc13 WT or C87G mutant or and analysed for substrate specific ISGylation by Western blotting in presence of reducing agent. Equal loading of total protein was verified by anti-alpha-tubulin immunoblotting. (D) FACS sorted GFP-positive HeLa cells from the experiment in (B) were lysed with and without 2-ME and analysed by Western blotting against endogenous MxA.

In order to confirm the different modes of ISG15 modification, we used a system for the transient downregulation of ISG15 by miRNA (Figure 7). As seen in Figure 7A, expression and excision of the mRNA coding for GFP and the miRNA against ISG15 resulted in a significant depletion of ISG15 in IFN-β induced cells in contrast to the expression of an mRNA coding for GFP and a control miRNA. We also established stable cell lines expressing these RNAs but in this setting, the expression of the miRNA was not high enough to suppress the strong induction of ISG15 by IFN-β (data not shown). Therefore we used the GFP expression to enrich transfected cells by FACS for Western blotting. In these cells, ISG15 expression was reduced to less than 25% compared to the control cells expressing the control miRNA (Figure 7B). Co-expression of the ISG15 knock-down construct with Ubc13 WT resulted in a strong reduction of the band above 34 kDa (Figure 7C). As shown before, this band and the presumed Ubc13 dimer were absent when the C87G mutant of Ubc13 was co-transfected. The ISG15 miRNA led also to a strong reduction of UBE1L ISGylation (Figure 7C). Furthermore we applied the miRNA approach to analyse the potential modification of MxA, which is strongly induced by IFN-β. Thus we could investigate the possible modification of transient transfected Flag-MxA (Figure 7C) as well as endogenous MxA (Figure 7D). Again we found no evidence for any kind of modification of MxA by ISG15.

## Discussion

In several studies, many proteins have been identified as substrates for ISGylation using proteomic approaches [24]–[26], [48]. Interestingly, not all substrates were found in every study. This could be explained by the use of different cell lines, different transfection of the ISG15 machinery as well as different ways of purifying ISG15 conjugates. Furthermore, only a small percentage of each individual substrate has been found to be ISG15 modified at any given time. We found that ISG15 modification of proteins is partially sensitive to reducing agents and that these conjugates also exist in living cells (Figure 1). The mutation of the single cysteine (Cys78) and the C-terminal di-glycine motif in ISG15 as well as the incubation of cell lysates with hydroxylamine showed that Cys78 forms disulphide bridges with other proteins. This residue is highly conserved within mammalian ISG15 genes and located in the hinge region between the two ubiquitin-like domains and has previously been shown to be highly reactive [43], [44].

We show that UBE1L, the E1 enzyme of ISG15, is a major substrate for ISGylation itself (Figure 3B) [24], [27].

Another ISG15 substrate that we analysed in more detail is Ubc13. Previously, ISGylation at lysine 92 of Ubc13 had been described to inhibit its function as ubiquitin conjugating enzyme [32], [45]. We found that Ubc13 can not only be modified by ISG15 via an isopeptide bond but it can also be modified via a disulphide bond between Cys78 of ISG15 and the single Cys87 of Ubc13 (Figures 4, 5, 7).

While we were able to confirm the ISGylation of both, Ubc13 and UBE1L, by transient downregulation of ISG15 with a specific miRNA we could not see any effect on the previously described substrate MxA (Figure 7). We also tried to isolate ISG15 modified MxA protein. Despite the use of several purification protocols, we could only observe additional bands of MxA in the absence of reducing agent. These bands were never positive for ISG15 in Western blotting nor were they reduced in ISG15 knock-down cells. This led us to conclude that these bands are caused by a SDS-PAGE artefact. Further, we also found no evidence for an ISG15 modification of hGBP1 (Figure 6A) or PML (Figure 6D).

In summary we conclude, that a large proportion of the ISG15 modifications in living cells are not attached to the substrates via the di-glycine motif but via disulphide bridges between the single cysteine in the hinge region of ISG15 and a cysteine of the substrate. Thus, it may be difficult to distinguish between these two types of modifications when immunoprecipitations or metal-chelate affinity purifications are applied to isolate ISGylated proteins, because usually little or no reducing agents are present. We show that mutagenesis of Cys78 and the C-terminal di-glycine motif can be used to discriminate these two possibilities. Furthermore, for a complete reduction of disulphide bridges and thioesters of ISG15, it seems to be essential to use SDS-loading buffer with freshly added and sufficient amounts of reducing agent.

The physiological relevance of the Cys78-dependent, atypical form of ISG15 modification remains unclear. For Cys87 in Ubc13 which is located in the active centre and is used to build thioesters with ubiquitin it is conceivable that a modification of this cysteine by ISG15 would as well inhibit the function of Ubc13 by preventing the loading with ubiquitin. We cannot exclude that cysteine-linked ISG15 modifications just represents an unspecific side reaction, but in principle it could also be regulated by the cellular redox potential or the concentration of reactive oxygen species due to cellular stresses. ISG15 Cys78 has already been shown to be highly reactive and is involved in dimerization which can be prevented by nitrosylation [43]. Recently, is has been shown that the classical ISGylation involving the isopeptide linkage formation targets mainly freshly synthesized proteins due to an association of the E3 ligase Herc5 with the ribosome [39]. The authors of this study concluded that ISGylation of only a proportion of viral proteins would impair the assembly of viral particles. This mechanism could also explain how ISG15 modification could act despite the substoichiometric modification of the substrates. Other studies have shown that the accumulation of ISGylated proteins is slow despite the rapid induction of ISG15 by IFN-β induction [21], [49] since the synthesis of the E1, E2 and E3 enzymes is delayed. Consistent with this, we found after induction of cells with IFN-β that the reduction sensitive conjugates occur several hours earlier than the insensitive ISGylated proteins (Figure 1E). Furthermore, also unconjugated ISG15 has been shown to have antiviral activity [4], [5]. Therefore another attractive hypothesis would be that the modification via the cysteine of ISG15 allows it to broaden its target scope and to achieve a similar amount of modified proteins before the synthesis of the ISGylation enzymes has been upregulated.

## Materials and Methods

### Plasmids and Antibodies

The cDNAs encoding for ISG15 (IMAGE ID IRAUp969E1149D, GenBank identification number (GI): BC009507), UbcH8 (UBE2H) (IMAGE ID IRAUp969G0731D, GI: BC006277), UBE1L (UBA7) (IMAGE ID IRAUp969H0433D, GI: BC006378), Ubc13 (IMAGE ID IRATp970B041D, GI: BC003365) and MxA (IMAGE ID IRATp970B1055D6, GI: BC032602) were purchased from ImaGenes (now Source BioScience). The pCDNA-Flag-Herc5 construct was kindly provided by K. Hochrainer [50]. The pCDNA4/TO/RGSHis_6_-SUMO2 has been previously described [47].

In order to generate expression vectors, ORFs were amplified by PCR and subcloned into the pTriEx-2 vector (Novagen), pCMV vectors (Stratagene) or modified pCMV2b vectors where the Flag-tag was replaced by either a double HA- or RGS-His-tag. ISG15 C78G, C78S and ΔGG as well as Ubc13 C87G mutations were obtained by site-directed mutagenesis using the Quik-Change Site Directed Mutagenesis kit (Stratagene).

Immunoblotting was performed with the following antibodies: mouse monoclonal anti-α-tubulin, clone DM1A (Sigma-Aldrich), rat monoclonal anti-HA, clone 3F10 (Roche), mouse monoclonal anti-FLAG M2 (Sigma-Aldrich), mouse monoclonal anti-penta-His (Qiagen), mouse monoclonal anti-RGS-His (Qiagen), mouse monoclonal anti-S-tag (Novagen), rabbit polyclonal anti-PML (Bethyl Laboratories) and rabbit polyclonal anti-ISG15 generated using recombinant ISG15 as antigen as described below. Mouse monoclonal anti-MxA (M143) was kindly provided by O. Haller and G. Kochs (University of Freiburg) [51]. Rat monoclonal anti-hGBP1 was kindly provided by M. Sturzl [52].

### Cell Culture and Transfection

HeLa B cells (ECACC # 85060701) were cultured in Dulbecco’s modified Eagle’s medium (Gibco) supplemented with 10% heat-inactivated calf serum (Sigma-Aldrich), penicillin (100 units/ml) (Gibco), streptomycin (100 µg/ml) and Non-Essential Amino Acids Solution 10 mM (100x) (Gibco) at 37°C with 5% CO_2_ in a humidified incubator. Cells were transiently transfected with GeneJuice (Novagen) according to the manufacturer’s instructions. For co-transfections, the total plasmid amount for each transfection was normalized by the addition of appropriate amount of empty vectors. For cytokine stimulation, 1,000 units/ml of human IFN-β (PeproTech) was used.

### Metal-chelate Purification of ISG15 Conjugates

Purifications of ISG15 conjugates were performed with or without reducing agent (DTT or 2-ME). In both cases, purification was carried out under strong denaturing conditions, which decreases unspecific binding to metal-chelate matrices. Cultured HeLa cells (4×10^6^) were transiently transfected with His-tagged ISG15 (pCMVb-MRGS-His-ISG15, pTriEx2-His-S-ISG15 wild type or its mutants) and other plasmids (pTriEx2-His-S-UBE1L, pCMV2a-Flag-UBE1L, pCMVb-HA-UbcH8, pTriEx2-His-S-UbcH8, pCMV2b-Flag-MxA, pCMVb-HA-MxA, pCMVb-HA-Ubc13, pCMVb-HA-Ubc13 C87G), in conformity with the experiment. 24 h post-transfection the cells were induced (if necessary) with IFN-β (1,000 units/ml) for 24 h. Before lysis, cells were rinsed twice with ice-cold PBS and cells were scratched with a cell scraper in 5 ml ice-cold PBS. The cell suspension was pelleted by centrifugation with low speed (300×g) for 5 min. PBS was aspirated and 600 µl of urea buffer (50 mM Tris-HCl, pH 8.0, 8 M urea, 5 mM imidazole, 1% Triton X-100 (w/V), 150 mM NaCl, 1% glycerol (w/V), with or without 5 mM 2-ME, 20 mM NEM, 25 µg/ml of leupeptin, aprotinin and pepstatin) was added for resuspension of the cellular pellet. DNA was removed by sonication. Cellular debris was pelleted by centrifugation at 16,000×g for 10 min at 4°C. The clarified cellular lysate was added to 50 µl TALON metal affinity resin (Clontech) pre-equilibrated with urea buffer and incubated for 2 hours on a rotation platform at 4°C. Afterwards, the resin was washed 5 times with urea buffer, 3 times with Washing buffer (the same as urea buffer but with 10 mM imidazole, pH 6.0). Finally, the resin was transferred to centrifuge filter systems Mini (Roth) and centrifuged with 2,000×g for 30 s at 4°C. The elution of ISG15 conjugates was performed with 2×SDS sample buffer containing 4 M urea, 50 mM EDTA, 750 mM imidazole and 500 mM 2-ME or not.

### Transient Downregulation of ISG15 by miRNA

The miRNA oligomers (ISG15 miRNA: TGCTGCTCACTTGCTGCTTCAGG TGGGTTTTGGCCACTGACTGACCCACCTGACAGCAAGTGAG; control miRNA: TGCTGAAATCGCTGATTTGTGTAGTCGTTTTGGCCACTGACTGACGACTACACATCAGCGATTT) for the transient downregulation of ISG15 were purchased from Invitrogen and cloned following the BLOCK-IT Pol II miR RNAi Expression Vector Kit with EmGFP manual. The advantage of this system is the easy detectable selective marker GFP, which is coded within the same mRNA in an open reading frame in front of the miRNA. This primary RNA will be cleaved by the Drosha complex to yield the GFP mRNA and the ISG15 or control miRNA. Therefore, cells which are positive for a GFP signal can be easily analysed by flow cytometry or immunofluorescence. For this purpose, HeLa cells were transiently transfected with control miRNA or ISG15 miRNA vectors. 12 h post-transfection, the cells were induced with IFN-β (1,000 U/ml) for another 12 h. Then, the cells were trypsinized to yield a proper cell suspension and analysed by a Becton Dickinson FACScan (Becton Dickinson GmbH). The percentage of sorted green cells was ≥ 70% for each experiment.

### Fluorescence Microscopy

For fluorescence microscopy, cells were grown on cover slips, transfected and IFN- induced as described above. Cells were fixed in PBS, 3% (w/V) paraformaldehyde, permeabilized with PBS, 0.2% Saponin (w/V, Roth) and blocked with PBS 0.2% Saponin (w/V), 3% BSA (w/V, Fraction V, protease free; Roth). Primary rabbit polyclonal ISG15 and secondary Alexa-labeled (Alexa Fluor 647 donkey-anti-rabbit IgG, Molecular Probes, Invitrogen) antibodies were applied in blocking buffer. Cover slips were embedded in ProLong Gold antifade (Invitrogen) and examined using a Zeiss Axioplan2 fluorescence microscope.

### Western Blotting and Immunoprecipitations

For Western blotting, cells were lysed in urea buffer (50 mM Tris-HCl, pH 8.0, 8 M urea, 1% Triton X-100, 150 mM NaCl, 1% glycerol, 1 µg/µl leupeptin, 1 µg/µl aprotinin, 1 µg/1 µl pepstatin). Cell lysates were mixed in a 5∶ 1 ratio with loading buffer (250 mM Tris-HCl, pH 8.0, 25% (w/V) glycerol, 7.5% (w/V) SDS, 0.25 mg/ml bromphenol blue) without or with 500 mM 2-ME. Protein samples were then resolved on 10% or 12% SDS-PAGE, transferred onto an Immobilon-P PVDF membrane (Millipore) and analysed by respective antibody staining as indicated. The quantification of Western blots was performed with Photoshop CS3 (Adobe Systems, San Jose, CA).

For immunoprecipitations, the indicated antibodies were covalently bound to protein A-agarose beads (GE Healthcare) using dimethyl pimelimidate, (DMP) (Merck KGaA) as cross linker. Alternatively, mouse anti-FLAG M2 beads (Sigma-Aldrich) or rat anti-HA beads (clone 3F10) (Roche) were used when indicated. Equal amounts of IFN-β treated and/or transfected cells were lysed in RIPA (radioimmunoprecipitation assay) buffer: 50 mM Tris-HCl, pH 7.5, 150 mM NaCl, 1% Triton X-100 (w/V), 0.1% SDS (w/V), 0.5% sodium deoxycholate, 40 mM NEM, 25 µg/ml of leupeptin, aprotinin and pepstatin. Cellular debris was removed by centrifugation at 12,000×g. The supernatant was diluted 1∶10 with PBS and incubated for 4–5 h with protein A-sepharose-coupled antibodies. Immunoprecipitates were eluted from beads with elution buffer (200 mM Tris-HCl, pH 9.0 and 1% SDS) and analysed by Western blotting with the indicated antibodies.

### Production of Polyclonal Rabbit Antibodies against Recombinant ISG15

Rabbit immunization and serum collection was performed at BioGenes using purified human ISG15 (see below). The antiserum with the best reactivity toward ISG15 was harvested, aliquoted and stored at −80°C until further use.

### Expression and Purification of Recombinant ISG15

*E. coli* BL21 (DE3) containing a plasmid (pTriEx-2) with the gene coding for His-S-tagged ISG15 protein were grown in LB medium at 37°C up to mid-log phase (OD about 0.7 at 600 nm). At this point, expression was induced by addition of 100 µM IPTG at 30°C for 5 h. The cells were harvested and resuspended in 50 mM Tris–HCl, pH 6.5, 5 mM CoCl_2_, 2 mM 2-ME, 10 mM imidazole, 150 mM NaCl, EDTA-free protease inhibitor cocktail (Roche), and lysed by sonication. Cellular debris was removed by centrifugation (40,000×g) for 40 min. The clarified supernatant was applied to Q-Sepharose column pre-equilibrated with lysis buffer. The flow through from Q-Sepharose was applied to Co^2+^-IMAC column pre-equilibrated in the same buffer. For elution, an imidazole gradient from 10 mM to 500 mM was applied over 3 column volumes. The eluted fractions containing His-S-ISG15 were collected and applied to Superdex 75 equilibrated with 50 mM Tris–HCl, pH 6.5, 2 mM 2-ME and 150 mM NaCl. In order to remove the tag from His-S-ISG15, the protein containing fractions from gel filtration were collected and subjected to limited proteolysis by thrombin. Finally, the protein solution was applied again to Superdex 75 pre-equilibrated with 10 mM sodium phosphate, pH 6.5 and 150 mM NaCl. The fractions corresponding to monomeric untagged ISG15 were collected and analysed by SDS-PAGE.

## Supporting Information

Figure S1 **No decrease of ISG15 conjugates by hydroxylamine.** IFN-β induced HeLa cells were treated with or without NEM (pre- and post-lysis as in Figure 1B) and 0.2 M hydroxylamine (post-lysis). PVDF membrane was stripped and immunodecorated with anti-actin antibody (low panel).
(TIF)
