# Supplementary figures and images for: Covalent Protein Modification with ISG15 via a Conserved Cysteine in the Hinge Region
Source: PLoS One. 2012 Jun 5;7(6):e38294. doi: 10.1371/journal.pone.0038294 (PMC3367918; doi:10.1371/journal.pone.0038294)

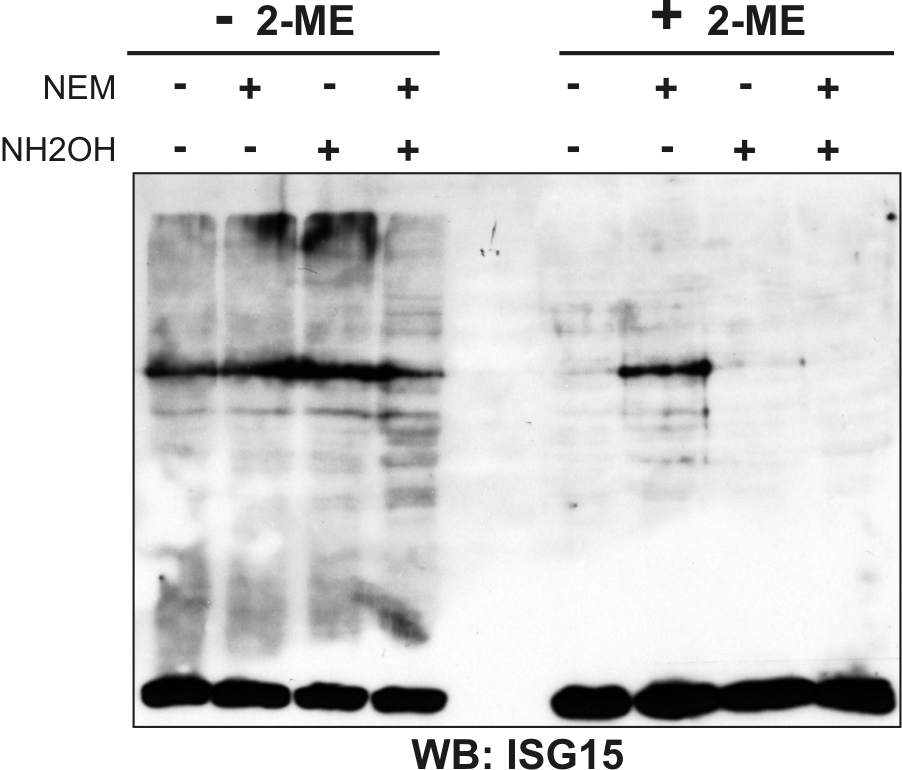

Supplement: Figure S1 — No decrease of ISG15 conjugates by hydroxylamine. IFN-β induced HeLa cells were treated with or without NEM (pre- and post-lysis as in Figure 1B) and 0.2 M hydroxylamine (post-lysis). PVDF membrane was stripped and immunodecorated with anti-actin antibody (low panel). (TIF) [file pone.0038294.s001.tif]
